# Reprogramming energy homeostasis in children with overweight through cognitive training and social interaction. A study protocol to estimate leptin sensitivity

**DOI:** 10.3389/fendo.2025.1606132

**Published:** 2025-06-18

**Authors:** Valentina Russo, Deny Menghini, Marco Mainardi, Danilo Fintini, Alessia Aureli, Nicoletta Gianni, Lucilla Ravà, Maria Alessia Rea, Gabriele Scozia, Chiara Spiezia, Gaia Scabia, Giulia Furini, Stefano Vicari, Stefano Cianfarani, Margherita Maffei, Melania Manco

**Affiliations:** ^1^ Child and Adolescent Neuropsychiatry Unit, Bambino Gesù Children’s Hospital, IRCCS, Rome, Italy; ^2^ Department of Biomedical Sciences, University of Padua, Padua, Italy; ^3^ Institute of Clinical Physiology, National Research Council, Pisa, Italy; ^4^ Endocrinology and Diabetes Unit, Bambino Gesù Children’s Hospital, IRCCS, Rome, Italy; ^5^ Research Unit for Preventive and Predictive Medicine, Bambino Gesù Children’s Hospital, IRCCS, Rome, Italy; ^6^ Clinical Epidemiology, Bambino Gesù Children’s Hospital, IRCCS, Rome, Italy; ^7^ Research Unit of Food Science and Human Nutrition, Department of Science and Technology for Sustainable Development and One Health, Campus Bio-Medico University, Rome, Italy; ^8^ Department of Life Science and Public Health, Catholic University of the Sacred Heart, Rome, Italy; ^9^ Department of Women’s and Children’s Health, Karolinska Institute and University Hospital, Stockholm, Sweden

**Keywords:** childhood obesity, childhood overweight, cognitive training, social training, environmental enrichment, leptin, leptin sensitivity, lifestyle modification

## Abstract

Leptin is a hormone secreted by adipocytes in proportion to the size of adipose tissue. By acting on its receptor, LepRb, primarily expressed in the hypothalamus, leptin promotes a negative energy balance by strongly suppressing appetite and stimulating energy expenditure. The majority of patients with obesity exhibit elevated leptin production, indicating that leptin becomes ineffective in regulating energy balance in this context. In the growing child, leptin modulates synaptic plasticity and thus leptin resistance may exert a key role in neurodevelopment. So far, studies in humans have investigated leptin secretion but not leptin resistance. We designed a research protocol (RESILIENT project) to study leptin sensitivity *in vivo* and ex vivo in growing children (6 to 11 years of age), and its association with neurodevelopmental features. The project will investigate the additive effects of Cognitive Training (CT) and Social Training (ST) on leptin sensitivity, compared to stand-alone Intensive Health Behaviour Treatment (IHBT) in children with obesity or overweight. *In vivo* investigation of leptin sensitivity may be particularly important for providing evidence of the metabolic and cognitive effects necessary for developing novel anti-obesity treatments.

**Clinical trial registration:**

https://clinicaltrials.gov/study/, identifier NCT06931730.

## 1 Introduction

Obesity is a complex chronic and relapsing disease caused by a combination of genetic, epigenetic, psychological, behavioural, social and environmental factors (1). According to the World Obesity Atlas 2024 report (2), the prevalence of childhood obesity has increased significantly over the recent years, with metabolic abnormalities already detectable in preschoolers at the onset of overweight (3). The prolonged COVID-19 lockdown, that has forced people to sedentary lifestyle, has negatively impacted health, leading to an increase in the prevalence of overweight and obesity, especially in youngers (4).

Children and adolescents with obesity are at an increased risk of experiencing depression, anxiety, social isolation, and impairments in self-esteem, self-efficacy, and quality of life (5–8), as well as sleep disorders (9). Additionally, cognitive deficits have been documented in this population (10), including impairments in long-term memory (11), executive functions (12), and attentional processes (13).

These negative effects appear to persist into adulthood, contributing to impaired synaptic plasticity and increasing vulnerability to age-related cognitive decline (14–16). Indeed, obesity is associated with brain alterations that affect individuals across the lifespan, from childhood to older adulthood (17). Among these, hippocampal abnormalities have been observed already in children with obesity (18). Hippocampal dysfunction may interfere with weight loss treatment (15) by weakening the ability to recall inhibitory associations tied to satiety signals (19). As a result, individuals may fail to regulate food intake effectively, responding instead to external food cues.

Leptin is a hormone that regulates appetite and energy expenditure by influencing energy homeostasis and satiety signalling. Leptin receptors are widely distributed in various brain structures, including the hippocampus, where leptin influences learning and memory processes (20, 21). Thus, leptin is involved in hippocampal long-term potentiation (22), long-term depression, and motivational eating (23). In individuals with obesity increased leptin secretion lead to leptin resistance, which represent a significant barrier to successful weight loss (15). Therefore, leptin resistance might be considered the molecular bridge between metabolic dysregulation and cognitive dysfunctions due to hippocampal abnormalities, particularly memory, often seen in obesity (24). In children, obesity-induced hyperleptinemia and leptin resistance may have an even greater impact on synaptic plasticity of their developing brain, contributing to emotional and behavioural dysfunctions (7) and cognitive deficits (10).

Current treatment strategies for overweight and obesity primarily focus on dietary modifications and Physical Activity (PA), both of which reduce body mass index (BMI) and improve metabolic parameters, including lipid profiles and blood sugar levels (25). However, given the multifactorial and complex nature of overweight and obesity, interventions (26) targeting cognitive abilities and social relationship - often reduced in individuals with excess weight—could provide significant benefits.

Cognitive Training (CT) may support early modulation of synaptic plasticity by mitigating leptin resistance, potentially contributing to weight loss. In animal models, the enhancement of cognitive and social stimulation, along with PA opportunities and sensorimotor stimulation, is encompassed within what is referred to as an enriched environment – EE (27). Specifically, studies in rodents have highlighted a significantly positive and protective effect of EE, influencing hippocampal neurogenesis as well (28). Moreover, while PA alone can partly restore leptin sensitivity (29), the enhancement of cognitive, sensory and social stimulation further increases leptin response, and causes a synaptic reorganization in hypothalamic feeding circuits (30). In a high-fat, high-cholesterol rat model, beneficial effects on cognition have been observed, alongside changes in gut microbiota composition. This suggests that energy homeostasis, cognition, and the microbiota are interconnected through the gut-brain axis (31). Additionally, in laboratory rodents, EE induces neural plasticity in brain regions responsible for metabolic homeostasis and cognitive function (32, 33), especially in early postnatal stages (30).

These findings suggest that comprehensive interventions, including cognitive and social stimulation, could be more effective in addressing obesity-related metabolic and cognitive dysfunctions, particularly in children and adolescents; however, evidence in humans remain limited (34, 35).

In summary, while traditional overweight and obesity intervention focus on diet and PA, multi-component interventions focusing on EE, which involve CT and Social Training (ST) may provide additional benefits by improving leptin sensitivity and enhancing neural plasticity.

This may be especially true in early childhood, which represents a critical time window to boost leptin sensitivity and reprogram the energy balance set point through appropriate protocols reproducing EE.

## 2 Hypothesis and aims of the resilient project

The present project hypothesizes that the combination of ST and CT with Intensive Health Behavior Treatment (IHBT; 26), which includes PA and dietary interventions, will lead to an improvement in leptin sensitivity in children following treatment for overweight and obesity.

Since direct assessment methods used in animal models are not feasible in humans, this study will rely on alternative proxies previously used to evaluate leptin sensitivity. In humans, except for rare pathological conditions such as congenital leptin deficiency or lipodystrophy, leptin administration is not approved. In rodent models, leptin sensitivity is typically assessed through a more direct and rigorous procedure. This involves administering recombinant leptin, followed by measuring food intake and/or evaluating leptin signalling in the brain. The latter is achieved by analysing the activation of intracellular effectors, such as STAT3 and Extracellular signal-regulated protein kinases 1 and 2 (ERK1/2), after hypothalamic dissection.

In our participants, we will estimate leptin anorectic effect by calculating the ratio between circulating leptin levels and total calorie intake measured during an ad libitum laboratory meal. This ratio will serve as the primary outcome to assess changes in leptin sensitivity. Additionally, molecular analysis will be conducted on Peripheral Blood Mononuclear Cells (PBMCs) to monitor the activation of leptin signalling pathways, as previously performed in other human studies (36).

As a secondary outcome, we will explore the association between changes in leptin sensitivity and improvements in cognitive measures, such as spatial and verbal long-term memory and spatial and verbal working memory.

Additionally, some supplementary endpoints will be measured at each time point to provide a more comprehensive understanding of the intervention’s effects.

## 3 Materials and methods

### 3.1 Study design

**Figure 1:**
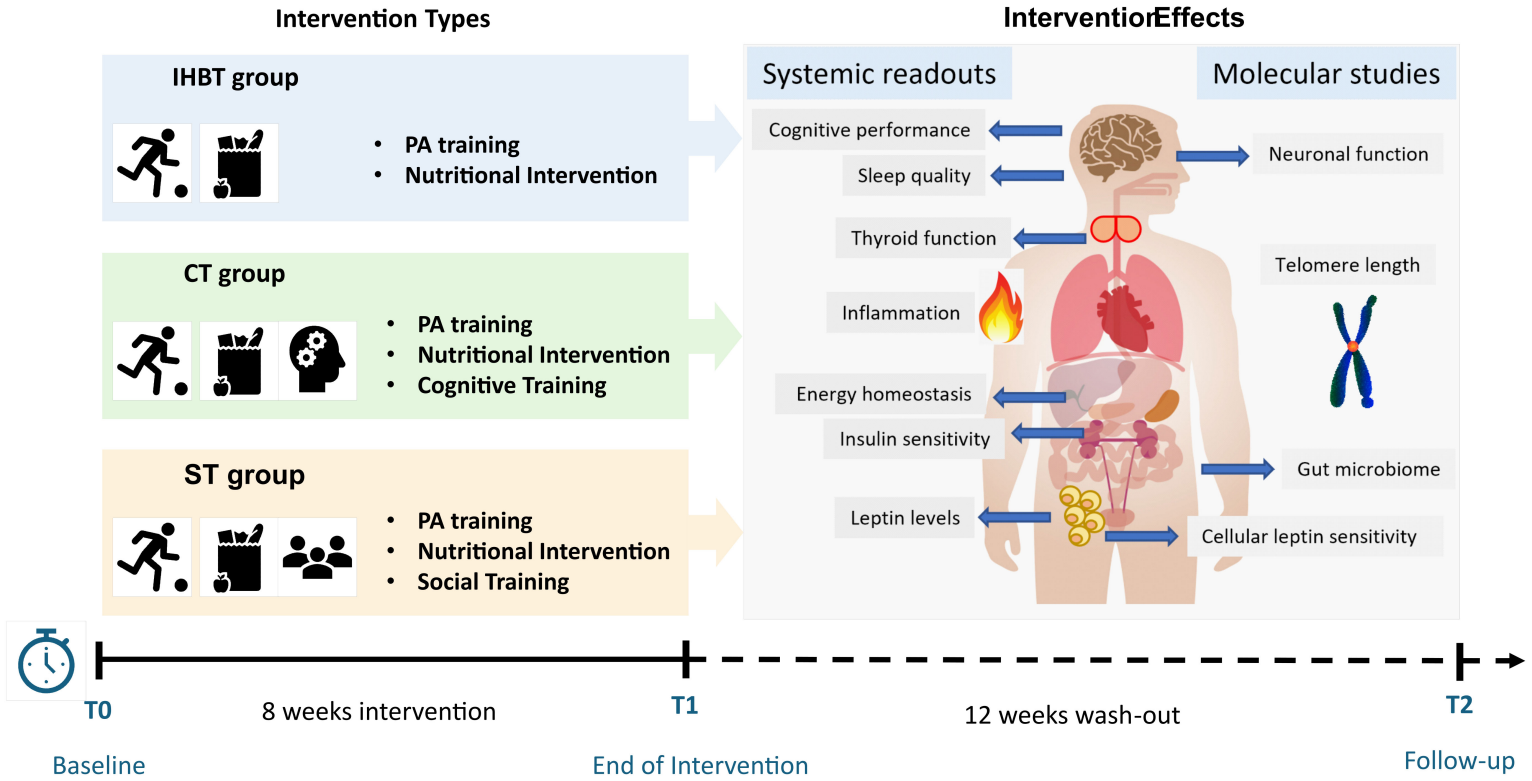
Study design and RESILIENT project timeline, showing the interventions types and intervention effects.

To test the hypothesis, we designed an 8-week randomized clinical trial (RCT) involving children aged 6 to 11 years with overweight or obesity. The choice to conduct an 8-week intervention was based on several considerations. Firstly, extending the intervention beyond this timeframe would likely reduce long-term participants compliance. Secondly, longer interventions require greater financial and personnel resources, making them less feasible in a clinical research context. Finally, although conducted in a murine model, the study the study by Mainardi and colleagues (30) demonstrated that relevant effects at the cellular and molecular levels are already observable after a similar duration, supporting the translational validity of our timeframe. Participants will be allocated to 1 of 3 groups: IHBT group, CT+IHBT group, and ST+IHBT group. After the intervention phase, all treatments will be suspended for a 12-week wash-out period to evaluate the persistence of effects on both primary and secondary outcomes. **Figure 1** summarizes the study design and RESILIENT project timeline.

### 3.2 Participants

A total of 120 children with overweight or obesity, referred to the Unit of Endocrinology and Diabetology of the Bambino Gesù Children’s Hospital in Rome, will be consecutively recruited during daily clinical activities by a team of highly trained endocrinologists and nutritionists and randomized to treatment. Parents or legal guardians will receive comprehensive instructions regarding the procedures and objectives of the experiment. Clinicians will obtain written consent and assent from the participating child before enrolment in the study. Participation will be solely voluntary.

No financial compensation or reward will be provided for participants.

Inclusion criteria are the followings: a) age 6 to 11 years-old; b) a condition of overweight and obesity defined as follows: *“overweight”* defined as BMI ≥ 85th percentile (1.036 SD) and < 95th percentile (1.645 SD) percentiles, *“obesity”* as a value of BMI ≥ 95th percentile (1.645 SD); and *“severe obesity”* as a value of BMI ≥ 120% of the 95th percentile (2.326 SD), based on CDC reference charts (37); d) an Intelligence Quotient (IQ) higher or equal to 85 (IQ ≥ 85); c) possession of electronic devices such as a computer or tablet.

Exclusion criteria include the presence of: a) genetic or syndromic obesity; b) reduced mobility; c) systemic diseases; d) ongoing pharmacological treatment for chronic conditions.

### 3.3 Trial arm allocation

Participants will be randomized into 3 8-week trainings as follows: a) IHBT group (n = 40); b) CT+IHBT group (n = 40) or c) ST+IHBT group (n = 40).

Randomization will be performed according to a block-randomization list, stratified by gender and age (6–11 years), and generated using the ralloc routine in STATA 17 software before enrolment.

### 3.4 Sample size

The sample size for the 3-arm study was determined considering the different decrease of leptin values (an indirect estimate of the primary endpoint leptin sensitivity), following the intervention (mean values of 10 mg/dL in the IHBT group compared to 6 mg/dL in the ST+IHBT or CT+IHBT group, SD = 4). An effect size of 1 was considered as clinically relevant (comparing CT+IHBT group vs. IHBT group and ST+IHBT group vs. IHBT group using Student’s t-test).

Pairwise comparisons will be performed with a significance threshold of p < 0.017 to account for multiple comparisons (3 comparisons), using Bonferroni corrections (p = 0.05/3). To achieve a significance level of alpha equal to 0.017 and a study power of 95%, 30 patients per group are required; considering a 30% dropout rate during follow-up, the plan is to recruit a total of 120 participants (40 per group). Missing data will be excluded from the statistical analyses; otherwise, if the proportion of missing values does not exceed 20%, we will use a mean substitution method, or a multiple imputation method (as for example KNN) as needed.

### 3.5 Interventions

All participants of the RESILIENT project will be provided with personalized dietary plan and a PA training as follows:

#### 3.5.1 Nutritional intervention

Participants will receive comprehensive dietary education from the nutritionists of the research team (NG and CS), aimed at improving their baseline diet by incorporating nutritional recommendations based on the Mediterranean diet and the Italian dietary guidelines for healthy eating (50). The education will include increased intake of vegetables, legumes, and fruits, along with reductions in energy-dense, nutrient-poor foods. To support these objectives, nutritional education will promote healthier eating behaviours across various aspects of daily life, such as establishing mealtime routines and encouraging family meals (26). Strategies will be tailored to align with individual preferences, family environments, and available support, ensuring that the nutritional approach is both effective and sustainable. Furthermore, parents will be actively engaged to support environmental changes and enhance compliance (38).

#### 3.5.2 Physical activity training

A kinesiologist (MAR) from the research team will provide participants with PA education to support the engagement in enjoyable and healthy daily activities, aiming to enhance motor competence and confidence. The kinesiologist will develop a structured exercise program tailored to the participant’s initial physical abilities and cardiorespiratory capacity, such as aerobic capacity, flexibility and coordination assessed at baseline. This approach seeks to improve physical fitness, enhance quality of life and support the achievement of age-appropriate physical levels. The PA training will be supervised by the kinesiologist over the 8-week period, with sessions held 2 to 3 times per week, depending on whether the participant practices sports. Each session will begin with a 10-minute warm-up and conclude with a 10-minute cool-down and stretching period, lasting approximately 60 minutes in total (26). The PA training will include weight-bearing and non-weight bearing games, aerobic, proprioceptive and resistance exercises, within a fun and supportive environment. All sessions will be conducted online. The decision to implement remote training is based on several considerations. Firstly, it aims to alleviate the burden and discomfort on the families, who would otherwise have to accompany the participant to a potentially distant gym, thereby reducing the risk of dropout. Moreover, conducting PA training in a gym setting could introduce social influences that may compromise the study’s validity, allowing at the same time the standardization of treatment across all participants. Lastly, during the COVID-19 pandemic, tele-exercise has demonstrated efficacy in managing paediatric obesity, facilitating behavioural changes, and promoting adherence to PA programs (39).

#### 3.5.3 Cognitive training

Participants will undergo an 8-week CT+IHBT program, 3 days a week, with each session lasting approximately 25 minutes. The training will be conducted using Cogmed (109), a commercial program that consists of a series of brief exercises that train cognitive functions such as attention and memory. The adaptive nature of the program ensures that the training intensity adjusts to each participant’s performance level, progressively increasing difficulty as they improve. The program’s interface is structured to be appealing and suited for school-age children, with brightly coloured elements. Correct responses are reinforced with positive visual cues (for example, lighter colours), while incorrect responses are signalled with negative ones (for example, darker colours). Additionally, as a reward for completing each training session, participants will be able to build and decorate their own *“island”*. Participants will use their own electronic devices, such as computer or tablet.

#### 3.5.4 Social training

A peer interaction program within an ecological setting, meant as a natural context of daily life (40), will be offered to children, who will participate in weekly sessions lasting 1.5 hours each. The program aims to improve active listening skills, comprehension of various communication styles (passive, assertive, aggressive), and the efficient recognition and management of emotions. The training will be supervised by trained psychologists (VR, GS) at the (110) (https://mdbr.it/en/who-we-are/mission/), in Rome. The activities that will be proposed to children align with the objectives of the RESILIENT project focusing on collaboration and communication skills, while avoiding cognitive tasks that could overlap with those administered in the CT+IHBT group. Additionally, these activities are designed to be sedentary to prevent the introduction of extra PA. Examples of the proposed activities include creative workshops focused on woodworking, paper crafts, weaving, and printmaking.

### 3.6 Study assessments

Outcome variables for all participants will be assessed at baseline (T0), at the end of the 8-week intervention (T1, end of month 2), and 12 weeks after the intervention (T2, end of month 5) to verify the persistence of effects. During the intervention period (T0-T1), participants will receive 3 online nutritional counselling sessions, each conducted every 15 days. Additionally, 2 online follow-up sessions (between T1-T2) are scheduled to occur once per month with nutritionists and PA trainer to support adherence to dietary guidelines, monitor progress, and provide ongoing support.

**Figure 2:**
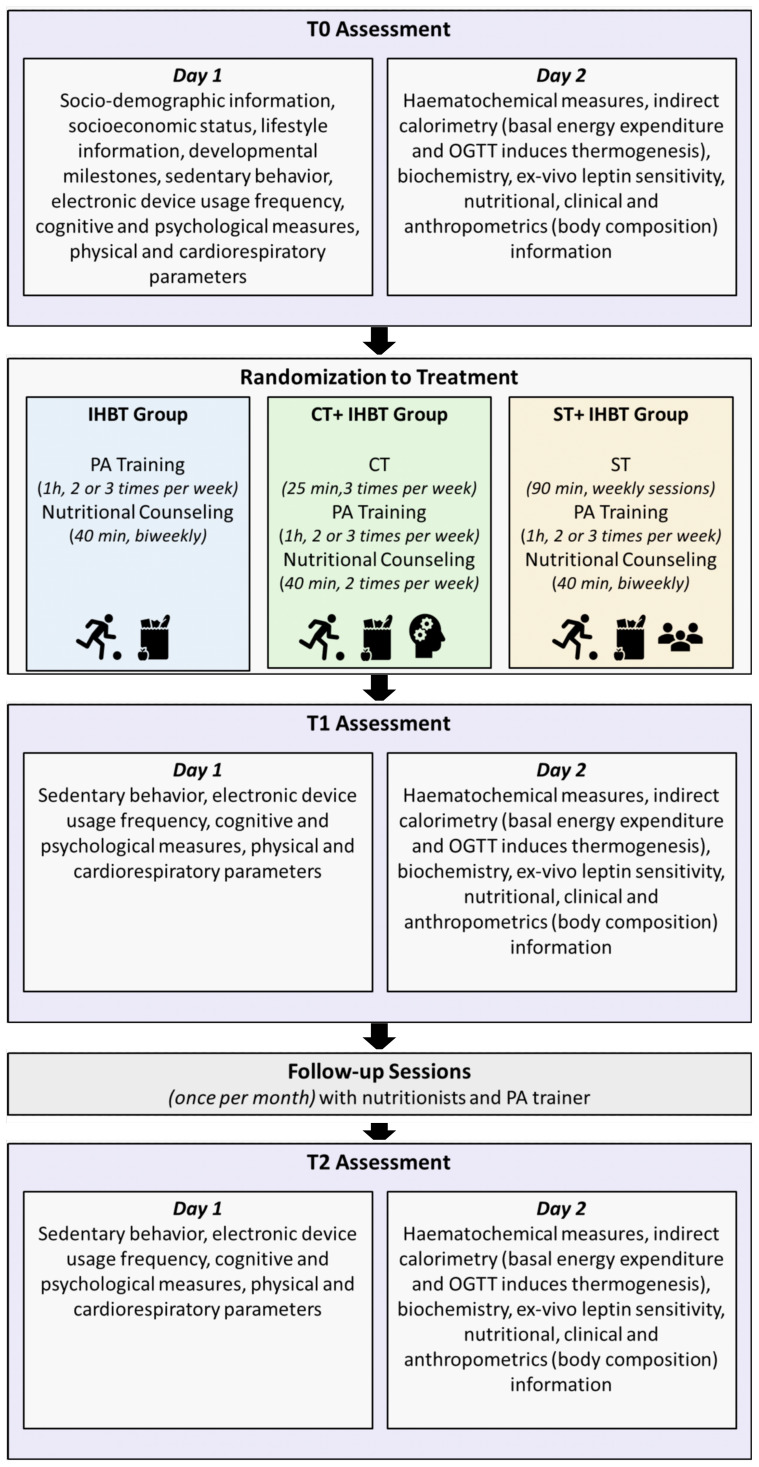
RESILIENT project procedure and assessments. Participants will undergo 3 assessment phases: baseline (T0), post-intervention (T1), and follow-up (T2). Each phase includes 2 days of testing. After the baseline assessment (T0), participants will be randomly assigned to one of 3 treatment groups (IHBT, CT+IHBT or ST+IHBT). All groups will receive nutritional counselling and PA training between T0 and T1. Additionally, one group will undergo cognitive training (CT+IHBT group), while the other group will undergo social training (ST+IHBT group). Monthly follow-ups will be conducted between T1 and T2.

**Figure 2** provides a detailed overview of the assessment and procedural steps involved in the study protocol across all phases of the research. Further details regarding the specific measures and evaluations conducted at each stage of the study will be extensively described in the subsequent sections of the manuscript.

At baseline (T0), all participants will undergo a comprehensive evaluation to collect socio-demographic information, including parents’ education, occupation and socioeconomic status. Data on each participant’s developmental milestones will also be gathered. Participants’ data will be recorded using the electronic health record.

To verify the efficacy of each training program (IHBT, CT+IHBT and ST+IHBT groups) a series of clinical, biochemical, psychological, cognitive and physiological measures will be assessed at each time point (T0, T1 and T2). Biological samples, including plasma, urine, faeces and PBMCs, will be collected and stored in accordance with good clinical practice.

The assessment will take place over 2 days: on the first day, cognitive, psychological, physical, and cardiorespiratory functions will be evaluated. On the second day, blood samples will be collected for haematochemical analysis, and indirect calorimetry will be performed to assess basal energy expenditure and OGTT-induced thermogenesis. Additionally, a comprehensive nutritional evaluation will be conducted.

All anthropometric and biochemical measurements will be conducted after an overnight fast, with participants instructed to avoid consuming any liquids for 4 hours before the analysis and to refrain from engaging in PA on the day prior to testing.

The integration of multiple assessment time points, combined with a randomized study design, will allow for a comprehensive evaluation of both short- and long-term effects of the intervention, enabling a comparative analysis of the different training programs.

#### 3.6.1 Physical and physiological assessments

Height will be measured with a Holtain stadiometer, while weight will be recorded using scales certified for medical use (90/384/EEC, SECA, Hamburg, Germany) with patients wearing minimal clothing. The average of 2 measurements will be used. BMI will be calculated using the standard formula: weight (kg)/height (m²), and the BMI z-score will be calculated using CDC reference values, expressed as SD score (37). Additionally, waist, hips and abdominal circumferences will be measured by non-elastic measuring tape. Body composition will be assessed through single-frequency Bioelectrical Impedance Analysis (BIA; DS Medica, BIA Light, Milano).

Indirect Calorimetry (COSMED, Q-NRG) will be conducted under fasting condition (8-hours fasting) for 15 minutes, with participants instructed to remain still, silent and awake during the test. Additionally, indirect calorimetry will be performed during the OGTT for 60 min, starting immediately after the oral glucose challenge (41). Resting Energy Expenditure (REE), Oxygen Consumption (VO2), Carbon Dioxide Production (VCO2), and Respiratory Quotient (RQ) will be recorded. Carbohydrate, lipid and protein oxidation, as well as Diet-Induced Thermogenesis (DIT), will be all estimated (42).

Biochemical analyses will include a complete blood count, blood lipids, liver and thyroid function tests, glucose, insulin and C-peptide, with the latter 3 measured at each OGTT time point using commercial kits.

Plasma samples will also be analysed for circulating levels of adipokines and cytokines (R&D Systems, Inc. 614 McKinley Place NE Minneapolis, MN 55413), including leptin, Brain-Derived Neurotrophic Factor (BDNF), a key regulator of neural plasticity and appetite suppression (43), Pro-OpioMelanoCortin (POMC) and its derivative Melanocyte-Stimulating Hormone (MSH), which play a central role in the anorectic response to leptin, eotaxin/CCL11, a proinflammatory chemokine linked to age-related cognitive decline (44), and Insulin-like Growth Factor-Binding Protein 2 (IGFBP2), a liver-derived protein regulated by leptin and involved in glucose homeostasis (45), also recognized as a marker of leptin sensitivity during weight loss (46).

Additionally, during the OGTT, acetylated ghrelin, Peptide YY (PYY), and Glucagon-Like Peptide (GLP) will be measured at each time point to further assess hormonal responses to glucose intake.

#### 3.6.2 Lifestyle assessment

Examiners will interview parents about their children’s lifestyle habits, including PA, sports participation, and sedentary behaviours, focusing on the frequency and duration of these activities. The assessment will also include detailed questions regarding children’s use of electronic devices, examining the weekly usage duration for each type of device, including television, computer, mobile phone, tablet, and gaming console. **Table 1** provides a brief overview of the tools that will be utilized to assess various lifestyle behaviours, including PA, sleep and eating habits.

**Table 1:** Brief description of tools that will be used to assess lifestyle behaviours including physical activity, sleep and eating habits.

Tool name	Constructs Assessed	Type	Outcome
Children’s Physical Activity Questionnaire (PAQ-C)	Frequency of sports and leisure activities, physical activity performed during the day (physical education classes, recess time, lunchtime, after school activities on weekday evenings and weekends)	Self-report	Raw Scores
International Physical Activity Questionnaire Short form (IPAQ-SF)	Moderate-intensity activities, vigorous-intensity activities, walking and sedentary habits, their frequency and duration on weekday and weekends	Parent-report	Raw Scores
The Sleep Disturbance Scale for Children (SDSC)	Disorders in initiating and maintaining sleep, sleep breathing disorders, arousal disorders, sleep–wake transition disorders, excessive sleepiness, and nocturnal hyperhidrosis	Parent-report	T-Scores
Eating Habits Questionnaire	Eating Habits	Parent-report	Qualitative Scoring
Food Diary	Eating Habits	Self-report	Qualitative Scoring
KIDMED	Adherence to Mediterranean Diet	Parent-report	Raw Scores

Motor and sedentary habits will be assessed using the self-reported Children’s Physical Activity Questionnaire (PAQ-C; 47), which measures moderate-to-vigorous PA levels over the past 7 days. The questionnaire consists of 9 items, rated on a 5-point Likert scale (1 = low activity, 5 = high activity), covering daily contexts. The overall score is calculated as the average of all items, excluding minus the last one, which serves as a qualitative indicator. Higher scores reflect greater levels of PA, while lower scores suggest a more sedentary lifestyle. Although no strict cut-off values are defined, raw scores allow for group comparisons and longitudinal tracking of PA levels.

During 2 online follow-up sessions (between T1-T2), the PA trainer will administer the parent-reported International Physical Activity Questionnaire Short form (IPAQ-SF; 48) for PA monitoring. The raw score will be calculated in MET-minutes/week by multiplying reported time spent in vigorous (8.0 METs), moderate (4.0 METs), and walking (3.3 METs) activities. Total MET-min/week determines 3 PA levels: low (< 600 MET-min/week), moderate (600 – 3.000), and high (> 3.000). Sedentary behaviour is recorded in minutes per day but will not contribute to the total MET score.

To assess sleep habits and quality, the Sleep Disturbance Scale for Children (SDSC; 49) will be employed. This standardized parent-report questionnaire evaluates various aspects of children’s sleep, including duration, onset time, sleep disorders and an overall measure of sleep disturbances in children and adolescents aged 6 to 15 years. Six main areas are investigated by the following subscales: Disorders in Initiating and Maintaining Sleep (DIMS), Sleep Breathing Disorders (SBD), Arousal Disorders (DA), Sleep–Wake Transition Disorders (SWTD), Excessive Sleepiness (DES), and Nocturnal Hyperhidrosis (NH). For each subscale raw scores will be converted in T-scores (M = 50, SD = 10).

Eating habits will be assessed through a combination of parent-report and self-report tools. Parents will complete a home-made eating habits questionnaire, while children will record their daily meals and snacks in a self-reported 7-day Food Diary. Both will be qualitatively analysed to provide insights into the child’s dietary patterns over the week. The evaluation will follow CREA (50) guidelines, including assessments of meal completeness, appropriate daily meal organization (5 meals-per-day), fruit and vegetable consumption (5 portions per day), age-adequate portions, and the frequency of weekly consumption of second courses (for example, meat, fish, legumes, eggs, cheese). Additional factors such as the absence of processed and industrially derived foods, sweets, and sweetened beverages, as well as water intake, will also be investigated. Macronutrient composition and calorie intake will be calculated from the Food Diary for comparisons during and after the intervention.

To assess adherence to the Mediterranean diet, the parent-reported KIDMED questionnaire (51) will be used. This 16-item measure assigns a score of -1, 0 or +1 to each item, resulting in a raw score ranging from 0 to 12 (< 3 = low adherence, 4-7 = medium adherence, > 8 = high adherence), referred to as the *“KIDMED score”*.

Finally, participants will receive in-person nutritional counselling with a nutritionist of the team to explore additional dietary habits and assess the presence of hyperphagia and voracity during meals.

#### 3.6.3 Physical and cardiorespiratory assessment

Physical exercise capacity will be assessed through a series of exercises focusing on cardiorespiratory fitness, joint mobility, flexibility, muscle strength, and balance. The aerobic power will be measured using a heart rate monitor, metronome, stopwatch and step counter (52). Joint mobility will be assessed through a Scapular-Humeral Circumduction test, conducted with a calibrated stick, where the result is the last completed circumduction with extended arms. Ankle dorsiflexion will be evaluated using the Knee to Wall test, where participants move their foot back until the knee touches the wall while keeping the heel on the ground. Column mobility will be measured manually with the Fingertip-To-Floor, assessing the distance of the fingers from the ground during forward bending (53).

Flexibility will be assessed using 2 tests: the Sit and Reach test, which measures the distance from the fingertips to the toes during forward trunk flexion, and the Lateral Trunk Flexion test, which records the difference between the initial hand position and the maximum flexion (54).

Muscle strength will be assessed through multiple tests: upper limb strength will be evaluated with a Floor Press test using calibrated dumbbells based on each participant’s strength, while handgrip strength will be assessed using a dynamometer. Lower limb explosive strength will be measured with the Standing Long Jump test, where participants jump as far as possible, landing with both feet together; the best of 2 attempts will be considered (54). Abdominal strength and endurance will be assessed with a Sit-Up test, where participants lie supine with their legs bent at 90° and perform sit-ups, with the result being the maximum number of repetitions performed in 60 seconds (55). Trunk strength will be assessed with a Trunk Lift test, where participants lie prone and lift their upper body to the maximum extent, with the chin-to-floor distance recorded (56).

Lastly, balance will be assessed using the Modified Clinical Test of Sensory Interaction and Balance Variant with drop counting, where participants maintain a single-leg stance within a shoe-sized rectangle for 60 seconds, with the number of falls recorded (57). The decision to utilize tests selected from various fitness test batteries was made to ensure a comprehensive, health-oriented evaluation of physical fitness, while also considering feasibility for both the participants and the setting of the project, while accounting for practical and age-related factors.

#### 3.6.4 Psychological assessment

Insights into various dimensions of the participants’ behavioural, psychological, emotional, and social functioning will be provided by both self-report and parent-report questionnaires, as summarized by **Table 2**.

**Table 2:** Questionnaires used to assess participants psychological domains.

Tool name	Constructs Assessed	Type	Outcome
Child Behavior Checklist for Ages 6–18 (CBCL 6–18)	Behavioural and emotional symptoms	Parent-report	T-Scores
Pediatric Quality of Life Inventory 4 (PedsQL version 4)	Physical, Emotional, Social, and School Functioning	Parent/self-report	Percentual Scores
Adaptive Behavior Assessment System-Second Edition (ABAS-II)	Social Adaptive domain	Parent-report	Standard Scores
Social Responsiveness Scale (SRS)	Social awareness, social cognition, social motivation and social communication	Parent-report	T-Scores
Eating Disorders Questionnaire in Childhood (EDQ-C)	Eating disorders, attachment patterns, anxiety and mood disorders, relational and behavioural difficulties	Parent/self-report (≥ 8 years old)	Standard Scores, Percentile Ranks

To investigate participants’ behavioural and emotional symptoms, the Child Behavior Checklist for Ages 6–18 (CBCL 6–18; 58) will be administered. The CBCL is a standardized parent-report questionnaire, comprised of 8 syndrome scales (anxious/depressed, withdrawn/depressed, somatic complaints, social problems, thought problems, attention problems, rule-breaking behaviour, and aggressive behaviour), 3 broadband scores (internalizing and externalizing problems, and total problem), DSM-oriented scales (affective problems, anxiety problems, somatic problems, attention deficit/hyperactivity problem, oppositional defiant problems, conduct problems), and the 2007 Scales (sluggish cognitive tempo, obsessive-compulsive problems, post-traumatic stress problems). For each subscale raw scores will be converted in T-scores (M = 50, SD = 10).

The Pediatric Quality of Life Inventory (PedsQL version 4; 59) is a widely used tool in studies involving children with obesity to measure key dimensions of health and quality of life, as outlined by the WHO (60). Developed through focus groups, cognitive interviews, pretesting, and field-testing (61), the PedsQL Generic Core Scales 4.0 includes domains of Physical, Emotional, Social, and School Functioning. Both child self-report and parent-report formats will be administered. Total Score and Psychosocial Health Summary Score will be calculated as percentages.

Adaptive skills will be assessed using the standardized parent-reported Adaptive Behavior Assessment System, Second Edition (ABAS-II; 62). In particular, the social skill domain will be investigated through the Social Adaptive Composite (SAC), which summarizes performance across the Leisure and Social skill areas. Raw scores will be converted into standardized weighted scores (M = 10, SD = 3) and standard scores (M = 100, SD = 15).

The Social Responsiveness Scale (SRS; 63) is a standardized parent-report measure frequently used with children and adolescents between the ages of 4 and 18 years (64) to screen subtle social communication deficits. The SRS generates 5 subscales: social awareness, social cognition, social motivation and social communication. Raw scores will be converted to gender-normed T-scores (M = 50, SD = 10).

The Eating Disorders Questionnaire in Childhood (EDQ-C; 65) is a standardized tool used for the early identification of eating disorders in children, assessing comorbidities with attachment patterns, anxiety and mood disorders, as well as relational and behavioural difficulties. It includes a self-report version for children aged 8 and older, and 2 parent-report forms based on the child’s age (4–7 and 8–12 years). For each diagnostic category, raw scores will be calculated and converted into standard score and percentile ranks (< 90 = within normal range, > 95 = clinically significant, > 99 = pathological).

#### 3.6.5 Neuropsychological assessment

The Colored Progressive Matrices (CPM; 66) will serve as a measure of non-verbal fluid intelligence due to its minimal reliance on language skills. The global Intelligence Quotient (IQ) will be considered in the analysis (M = 100, SD = 15).

The N-back (67) is a computerized task testing both visuo-spatial and verbal working memory. In the visual-spatial condition, participants view a series of visual stimuli (blue boxes) positioned randomly on the screen. Following a training phase, participants must indicate whether the location of each box matches that of the preceding 1 (level: 1-back). When response accuracy reaches or exceeds 80%, the difficulty level of the n-back is increased (for example, advancing from 1-back to 2-back). Similarly, the verbal condition requires participants to listen to a continuous stream of letters and the goal is to decide whether each letter matches the 1 heard immediately before (level: 1-back). Exercise progression follows the same protocol as the visuo-spatial condition.

The Continuous Performance Test-II (CPT-II; 68) is a computerized standardized measure of sustained performance that provides indices related to the attentional domain as well as executive functions, such as inhibitory control. Participants are instructed to press the spacebar when any letter except the letter *“X”* appears on the screen. Reaction Time (RT) is measured from the point at which any letter other than *“X”* appears on the screen until the spacebar is pressed (Go trial). No-Go trials occur when an *“X”* is presented. To mitigate the impact of repeated testing familiarity, a 3-minute practice will be presented prior to starting the CPT-II. Accuracy, reaction times in milliseconds, and Reaction Time Variability (RTV) in milliseconds will be included in the analyses.

To assess episodic long-term memory, the Verbal and Visual-Spatial Learning tasks from the standardized Battery for the evaluation of Memory (PROMEA; 69) will be administered. The Verbal Learning Task consists of a list of 15 semantically unrelated words that is presented orally for 3 consecutive trials. After each presentation participants will be asked to immediately recall as many words as possible, in any order; the number of words recalled across the 3 trials will be scored. Fifteen minutes after the third trial, participant will be requested to recall as many presented words as possible; the number of elements correctly recalled will be scored. Similarly, in the Visual-Spatial Learning Task, 15 figures of common objects positioned within 4 quadrants are presented in the study phase. After a 1-minute interval begins the test phase, where the target stimuli are presented individually, and the participant is asked to indicate the correct position. The test is repeated 3 times and, as in the verbal task, the number elements correctly recognized across the trials will be scored. Fifteen minutes after the third trial the test phase only will be presented again to assess the delayed recognition, scoring the number of elements correctly recalled. For each index, raw scores will be calculated and converted into standard score and percentile ranks.

The Forwards and Backwards Digit Span and the Corsi Block-Tapping tests from the standardized Battery for Neuropsychological Evaluation of Children (BVN 5-11, 70) will be used to assess verbal and visuo-spatial short-term memory and working memory. In the Digit Span test participants are asked to repeat an increasing sequence of numbers, in the same or in the reversed order as presented by the experimenter. The score is determined by the number of items correctly recalled. A similar procedure is used with the Corsi Block-Tapping test, which consists of blocks on a wooden board. Participants have to observe sequences of blocks tapped by the experimenter and then immediately reproduce them, in the same or in the reversed order. Raw scores will be converted into z-scores based on the mean and standard deviation of the normative sample.

The Movement Assessment Battery for Children-2 (MABC-2; 71) will be used to assess participants gross and fine motor skills. This battery produces a total score and 3 component scores: a) the Manual Dexterity component evaluates fine motor skill; b) the Aiming and Catching component assesses the coordination of fine and gross motor skills; c) the Balance component assesses gross motor skills and includes a static balance task and 2 dynamic balance tasks. Raw scores will be converted into standard scores (M = 100, SD = 15) and percentile ranks.

In order to assess visual-motor skills the standardized Test of Visual Motor Integration (VMI; 72) will be employed. This test comprises 3 components: a) the Visual Motor Integration component, where children are required to imitate and copy a series of increasingly complex forms; b) the Visual Perception, that requires participants to identify matching forms when presented with similarly shaped forms; c) Fine Motor Coordination component, which involves children’s ability to connect dots and stay within lines of the forms. Raw scores will be converted into standard scores (M = 100, SD = 15) and percentile ranks.

#### 3.6.6 Leptin sensitivity assessment

##### 3.6.6.1 *In Vivo* measures

Leptin sensitivity will be assessed *in vivo* as the ratio between the amount of calories introduced during an experimental meal and the participant’s circulating leptin levels. Specifically, the nutritionists (NG and CS) will provide each participant with 2 meals (breakfast and ad libitum lunch), collecting data on eating behaviour, time of consumption, request for additional food, and portion sizes consumed. Blood samples for leptin sensitivity assessment will be collected in fasting condition, prior to the breakfast, at three different time points (T0, T1 and T2).For breakfast, participants will be given a choice of sweet, salted or mixed breakfast, whose composition will be based on foods commonly liked and eaten by children. The breakfast box will contain 273–363 kcal (representing 15-20% of the daily energy intake based on the average basal metabolic rate and total daily energy expenditure at 75th percentile for children aged 6–11 years old, as reported by Reference Intake Levels for Nutrients and Energy for the Italian Population (111). Macronutrient distribution will be 13-15% protein, 26-34% fat, and 53-58% carbohydrates, as previously described in the literature (73–75).

Children will be instructed to consume their breakfast within 30 minutes (73–75) and to stop once satiety is reached. After 3 hours, an ad libitum lunch will be served and consumed within 1 hour (73–75). At the end of both meals, any remaining food will be weighed using a kitchen scale to determine the participant’s actual energy intake. Nutrient intake (protein, fat, saturated fatty acids, carbohydrates, sugars, fiber, salt) will be recorded using the nutritional labels from each food item, as available on the corresponding brand websites.

Hunger and satiety will be assessed using Visual Analogue Scales (VAS) administered at different time points: before breakfast, immediately after breakfast, before the ad libitum lunch, after lunch and again 2 hours later (76). Prior to the VAS assessment, a brief training questionnaire will be provided to facilitate the children’s understanding of hunger and satiety (77), based on the sample rating scale by Bennett and Blissett (78), specifically adapted for primary school-aged children. The ratings provided by the children will be analysed by nutritionists and converted into numeric values (0-10).

By administering meals to children, it is possible to assess changes in satiety sensation. When comparing caloric and macronutrients intake at T0, T1 and T2, differences in food consumption - both in terms of quality and portions sizes - will be analysed. This will help determine the effectiveness of nutritional intervention during the first 8 weeks and the maintenance period.

##### 3.6.6.2 Ex Vivo measures

Since direct assessment of leptin sensitivity in the central nervous system is not feasible in humans, we will evaluate peripheral leptin sensitivity ex vivo using PBMCs. Lymphocytes express the active form of the leptin receptor, and their responsiveness to leptin has been used as a proxy for central leptin sensitivity (36). The use of this proxy is further supported by evidence from animal studies, where a lower value of the food intake/leptin levels index correlates well with more direct measures of leptin sensitivity. These include detailed assessments of food intake following exogenous leptin administration and evaluations of leptin signalling, such as the expression of phosphorylated STAT3 in the arcuate nucleus of the hypothalamus (30, 79, 80).

PBMCs will be collected from the same individuals before and after the intervention and exposed to recombinant leptin to assess intracellular signalling activation. The primary leptin effector pathway is JAK2-STAT3, though ERK1/2 activation has also been reported among others (81). The activation of these pathways will be measured using immunoassays such as Enzyme Linked Immunosorbent Assay (ELISA) and Western blot, quantifying the ratio of phosphorylated to total protein levels for each intracellular effector. To mitigate the potential bias arising from inter-individual variability in lymphocyte proportions, we will normalize our signalling data by the lymphocyte counts obtained from each participant’s complete blood count with differential. Specifically, we will express our results as [e.g., fold change relative to baseline per 10^6 lymphocytes] or include the lymphocyte percentage as a covariate in our statistical models to account for this compositional variation. This approach will allow us to better discern the effects of leptin despite the use of a heterogeneous PBMC population.

Therefore, in addition to investigating the activation of STAT3, the main intracellular effector of leptin signalling, our research plan will analyse: 1) the PI3K–Akt Pathway, a major molecular pathway associated with metabolic regulation, insulin signalling, glucose homeostasis, and neuroendocrine function (82); 2) the AMPK pathway, which is known to be activated directly by leptin in peripheral tissues (83) but inhibited in the hypothalamus; and 3) the function of SOCS3, a negative regulator of the JAK2-STAT3 signalling pathway relevant to the leptin resistance state (84). We will aim to assess protein expression and phosphorylation of key components within these pathways in response to our interventions.

## 4 Discussion

The strengths of the RESILIENT project are its ground-breaking hypothesis, which proposes that CT+IHBT and ST+IHBT could synergistically enhance IHBT, increasing effectiveness of diet and PA in addressing obesity-related dysfunctions.

This hypothesis builds on the growing understanding that obesity is a multifactorial condition influenced by genetic, environmental, behavioural, and psychological factors (1). Depression, social isolation, impaired self-esteem and quality of life, as well as cognitive deficits including long-term memory, are frequently seen among individuals with obesity, even among children (5–7).

Obesity has been increasing over the years, especially during the COVID-19 pandemic (4, 85), which has further diminished positive exposure to environmental, social, sensory, and cognitive stimuli. Throughout this sedentary period characterized by increased screen time, there was a noticeable rise in the occurrence of psychosomatic and psychological symptoms (86), with impairments in decision-making including dietary choices. The elevated stress levels during this period caused individuals, particularly those with obesity, to adopt unhealthy eating habits (87). This trend is particularly concerning among children (88), who may experience early metabolic abnormalities (3) and obesity-related brain alterations, such as impaired hippocampal function, that can interfere with weight loss treatments (15, 89–91).

Negative effects of poor diet and obesity persist throughout adulthood, leading to cognitive decline and an increased risk of dementia in later life (15). These deficits in the adults can be connected to the loss of responsiveness to the neurotrophin-like effects of leptin on hippocampal neurons displayed by young mice fed a high-fat diet (92).

The early management of obesity is crucial, as childhood represents a critical period for behaviour pattern formation and the development of self-regulation abilities, which enhances the likelihood of maintaining healthy lifestyle habits throughout life (93).

Lifestyle modifications, including PA, diet, cognitive, and social stimulations, could be the key to achieving long-lasting treatment effects. Several studies indicate that children growing up in EE, with access to cognitively stimulating activities, are less likely to develop obesity (94). EE provides a combination of social, motor, cognitive, and somatosensory stimulation (95). In animal models, exposure to EE induces both structural and functional brain changes, enhancing learning and memory, increasing long-term potentiation, facilitating neurotrophin expression, boosting adult neurogenesis and brain neuroplasticity (96, 97). EE also slows the progression of neurodegenerative disorders and reduce brain inflammation in both animal models (44) and in elderly humans with Mild Cognitive Impairment (98). In this regard, EE-like protocols have also been found to reduce peripheral levels of eotaxin in elderly humans and aged mice. Eotaxin is a proinflammatory chemokine that negatively affects hippocampal neurogenesis and cognitive performance (44, 99). These findings are particularly relevant since brain inflammation has already been observed in adolescents with obesity and peripheral insulin resistance (100, 101).

Moreover, research suggests that EE increases leptin sensitivity, enhancing treatment outcomes (30). Leptin plays a neuroprotective role (102, 103), potentially slowing the progression of neurodegenerative disorders (104). Lower plasma leptin levels have been reported in individuals with mild cognitive impairment or Alzheimer’s Disease compared to controls (105). Leptin affects brain regions such as the hippocampus, modulating synaptic plasticity and cognitive functions like long-term memory (20, 21, 32). By linking metabolic regulation to cognition, leptin’s role in obesity-related hippocampal abnormalities suggest that it may be the biochemical link connecting cognition and weight-gain/loss. Notably, EE has been shown to programme hypothalamic leptin sensitivity most effectively when performed within a critical juvenile period (30), suggesting that a similar approach should be adopted in humans with obesity or overweight. Accordingly, multidiscipline lifestyle interventions in children with obesity have already shown promising effects leading to weight loss and reducing the prevalence of metabolic syndrome (38, 106, 107). Although the long-term efficacy of lifestyle interventions remains unclear, parental engagement is essential for achieving successful and sustained behavioural change (108). To this end, the RESILIENT project includes a multidisciplinary team that will conduct periodic assessments and counselling sessions to enhance parental awareness and promote adherence.

In a society increasingly deprived of social stimuli, relying solely on calorie-reduction diets or paediatric medications may overlook key factors. These include motivation, social adaptation, and functional decision-making, which are essentials for long-term success and well-being.

The present study could provide the first evidence in children that EE has additive effects beyond PA alone in reversing leptin resistance in overweight or obesity. It will also allow to delineate the distinct roles of CT, ST, and PA in treatment success. Moreover, it will provide insight into the molecular mechanisms downstream of the leptin pathway that modulates the interaction between energy homeostasis and neurocognitive function.

An additional unique feature is that the present study will provide and validate new tools to investigate leptin sensitivity *in vivo* in humans. Such approach will provide both molecular insights and innovative research procedures to study brain metabolism in conditions of systemic insulin resistance and low-grade inflammation. In fact, there isn’t currently a gold standard method for effectively assessing leptin sensitivity in humans, or a clear criterion established for the diagnosis of leptin resistance.

Moreover, RESILIENT project will offer a protocol for the study *in vivo* and ex vivo of leptin sensitivity that can be reproduced for evaluating metabolic and cognitive effects of anti-obesity medications, i.e. not just glucagon like peptide 1 and setmelanotide, but also amylin.

A key strength of this project lies in its multilevel assessment and intervention approach, which seamlessly integrates psychological, cognitive, neurophysiological, and physiological dimensions.

This comprehensive approach is essential to evaluate and treat overweight and obesity, inherently complex conditions (1), for which the most suitable intervention is a multicomponent lifestyle approach. Identifying which components of lifestyle interventions are most beneficial for specific domains could optimize the outcomes of obesity and overweight treatment programs, potentially leading to more sustainable and effective results.

The present study is part of a larger project aimed at examining therapeutic responses across different age groups, with a particular focus on young adults and the elderly.

By applying tailored interventions, including PA, ST+IHBT, and CT+IHBT, the project seeks to identify whether specific life stages present optimal windows to influence leptin sensitivity and the mechanisms underlying the energy homeostasis set point.

Additionally, RESILENT project provides an opportunity to validate these findings in clinical settings, particularly in children. Early interventions against obesity capitalize on the heightened plasticity of the brain and other organs during childhood, offering the potential for permanent, long-lasting effects into adulthood. On this account, a notable aspect of this project is its focus on creating new opportunities for early-life interventions and prevention. Given the increasing need for effective strategies to manage overweight and obesity from a very early age, this approach may provide substantial public health benefits.

In sum, the findings of RESILIENT project could significantly impact obesity prevention and treatment strategies, leading to more cost-effective prevention strategies, higher treatment success rates, and enhanced support for individuals dealing with obesity-related stigma and social disparities through CT and ST.

## Glossary

ABAS-II: Adaptive Behavior Assessment System, Second Edition
BDNF: Brain-Derived Neurotrophic Factor
BIA: Bioelectrical Impedance Analysis
BMI: Body Mass Index
BVN: Battery for Neuropsychological Evaluation of Children
CBCL: Child Behavior Checklist for Ages 6–18
CDC: Disease Control and Prevention
CPM: Colored Progressive Matrices
CPT-II: Continuous Performance Test-II
CT: Cognitive Training
DA: Arousal Disorders
DES: Excessive Sleepiness
DIT: Diet-Induced Thermogenesis
EDQ-C: Eating Disorders Questionnaire in Childhood
ERK1/2: Extracellular signal-regulated protein kinases 1 and 2
EE: Enriched Environment
ELISA: Enzyme Linked Immunosorbent Assay
GLP: Glucagon-Like Peptide
IGFBP2: Insulin-like Growth Factor-Binding Protein 2
IHBT: Intensive Health Behaviour Treatment
IPAQ-SF: Physical Activity Questionnaire Short form
IQ: Intelligence Quotient
MABC-2: Movement Assessment Battery for Children-2
MET: Metabolic Equivalent Task
MSH: Melanocyte-Stimulating Hormone
NH: Nocturnal Hyperhidrosis
OGTT: Oral Glucose Tolerance Test
PA: Physical Activity
PAQ-C: Children’s Physical Activity Questionnaire
PBMCs: Peripheral Blood Mononuclear Cells
PedsQL: Pediatric Quality of Life Inventory
POMC: Pro-OpioMelanoCortin
PROMEA: Battery for the evaluation of Memory
PYY: Peptide YY
RCT: Randomized Clinical Trial
REE: Resting Energy Expenditure
RQ: Respiratory Quotient
RT: Reaction Time
RTV: Reaction Time Variability
SAC: Social Adaptive Composite
SBD: Sleep Breathing Disorders
SDSC: Sleep Disturbance Scale for Children
SRS: Social Responsiveness Scale
ST: Social Training
SWTD: Sleep–Wake Transition Disorders
VAS: Visual Analogue Scales
VCO2: Carbon Dioxide Production
VMI: Test of Visual Motor Integration
VO2: Oxygen Consumption
